# A comparative study in left-sided breast cancer treated with moderate deep inspiratory breath hold versus free breathing

**DOI:** 10.1186/s43046-024-00214-6

**Published:** 2024-04-08

**Authors:** Anupam Muraleedharan, Sandip Kumar Barik, Deepak Kumar Das, Saroj Kumar Das Majumdar, Bikash Ranjan Mahapatra, Bijay Kumar Barik, Mathan Kumar Ramasubbu, Nehla Haroon K. M., Poornima Devi U., Sk Soel Ahmed, Priyanka Mukherjee, Ashutosh Pattanaik, Avinash Badajena, Minakshi Mishra, Satyabrata Kanungo, Sovan Sarang Dhar, Dillip Kumar Parida

**Affiliations:** 1 Department of Radiation Oncology, All India Institute of Medical Sciences, Bhubaneswar, India; 2 Department of Radiation Oncology, Utkal Hospital, Bhubaneswar, India; 3 Department of Pharmacology, All India Institute of Medical Sciences, Bhubaneswar, India; 4 Department of Radiation Oncology, All India Institute of Medical Sciences, New Delhi, India; 5 Department of Radiation Oncology, All India Institute of Medical Sciences, Mangalagiri, India; 6 Department of Radiation Oncology, Apollo Hospital, Bhubaneswar, India; 7 Department of Radiation Oncology, SUM Ultimate Medicare, Bhubaneswar, India; 8 Department of Radiation Oncology, All India Institute of Medical Sciences, Patna, India; 9 Department of Radiation Oncology, PGIMER and Capital Hospital, Bhubaneswar, India; 10 Department of Radiation Oncology, Lady Hardinge Medical College, New Delhi, India

**Keywords:** Breast cancer, Radiotherapy, 3D CRT, ABC, mDIBH, Cardiac sparing

## Abstract

**Background:**

The moderate deep inspiratory breath hold (mDIBH) is a modality famed for cardiac sparing. Prospective studies based on this are few from the eastern part of the world and India. We intend to compare the dosimetry between mDIBH and free-breathing (FB) plans.

**Methods:**

Thirty-two locally advanced left breast cancer patients were taken up for the study. All patients received a dose of 50 Gy in 25 fractions to the chest wall/intact breast, followed by a 10-Gy boost to the lumpectomy cavity in the case of breast conservation surgery. All the patients were treated in mDIBH using active breath coordinator (ABC). The data from the two dose volume histograms were compared regarding plan quality and the doses received by the organs at risk. Paired *t*-test was used for data analysis.

**Results:**

The dose received by the heart in terms of V5, V10, and V30 (4.55% vs 8.39%) and mean dose (4.73 Gy vs 6.74 Gy) were statistically significant in the ABC group than that in the FB group (all *p*-values < 0.001). Also, the dose received by the LADA in terms of V30 (19.32% vs 24.87%) and mean dose (32.99 Gy vs 46.65 Gy) were significantly less in the ABC group. The mean treatment time for the ABC group was 20 min, while that for the free-breathing group was 10 min.

**Conclusions:**

Incorporating ABC-mDIBH for left-sided breast cancer radiotherapy significantly reduces the doses received by the heart, LADA, and left and right lung, with no compromise in plan quality but with an increase in treatment time.

## Background

Breast cancer is the most common cancer diagnosed worldwide and in India [1, 2]. In India, the burden of breast cancer was pegged at 2.05 lakhs cases in 2020, projected to reach 2.32 lakhs by 2025 [3]. It remains the fourth most common cause of cancer death [1, 2].

Based on the clinical extent and pathologic characteristics of tumours, the management of breast cancer optimally is in a multidisciplinary setting. Surgery, chemotherapy, targeted therapy, and radiotherapy form the basis for the management of breast cancer. For patients with breast cancer who have undergone surgery, postoperative irradiation of the whole breast and chest wall with or without peripheral lymphatics are indicated in patients with high-risk characteristics, positive lymph nodes or positive resection margins.

It is well documented that the tangential radiotherapy fields used for breast cancer treatment can lead to irradiation of cardiac tissue in left-sided more than right-sided breast cancer radiotherapy [4]. Such incidental irradiation can lead to cardiac changes in the form of pericardial or myocardial fibrosis, valvular abnormalities, or coronary vascular damage [5].

Mortality from heart disease was significantly increased by 27% in patients receiving radiotherapy, most owing to coronary artery disease. Any increment in dose to the heart during radiotherapy will increase the rates of major coronary events, particularly in the case of the left breast [6].

Current treatment standards prescribe the use of drugs such as anthracyclines, trastuzumab, and aromatase inhibitors in the chemotherapy setting for breast cancer. Since these drugs have inherent cardiovascular risks associated with their use, it becomes even more critical to reduce cardiac exposure to radiation to the least possible level. Older techniques of cardiac sparing, such as the usage of heart blocks and prone position breast boards, had their limitations, which led to the advent of respiratory motion management strategies, of which active breathing coordinator is a promising tool.

Active breath coordinator is a device t synchronizes radiation delivery in breast cancer patients with the respiratory cycle. This device uses the normal physiologic inferior and posterior-medial movement of the heart when a patient takes and holds a deep breath and only delivers radiation when the patient is in specified portions of the breath-hold cycle. This can potentially reduce the irradiated volume and the dose to the heart and other thoracic structures.

Even though the mDIBH technique using active breathing coordinator (ABC) has been in vogue since the first decade of the millennium, prospective studies based on this modality are few from the eastern part of the world and India.

## Method

The study is a prospective observational study to compare the dosimetry between mDIBH and free-breathing (FB) plans. Between December 2021-November 2022, all patients with left-sided carcinoma breast requiring radiation therapy were included in the study with the following criteria: (A) left-sided breast cancer patients aged 18–70 years, (B) Eastern Cooperative Oncology Group performance score 0–2, and (C) able to understand the technique of breath holding and comfortably able to breath hold for a duration of 20–25 s. Patients with previous history of radiotherapy to the breast, cardiac and lung diseases, and history of chest wall injuries/anomalies were excluded from the study.

A sample size of 32 was found to achieve a power of 85% to detect a difference of 300 cGy in dose between the two treatment plans with a standard deviation of 400 cGy and a level of significance of 0.05. After establishing the diagnosis of breast carcinoma, 32 patients were included in the study after confirming the inclusion and exclusion criteria. The patient was explained about the ABC technique, and informed consent was taken for their participation in the study. Post surgery (mastectomy/breast conservation surgery), the patients underwent their scheduled chemotherapy regimens followed by adjuvant radiotherapy by ABC-mDIBH. Anti-Her2neu therapy was given wherever indicated. Post completion of radiotherapy, patients who were hormone receptor positive received hormonal therapy. They were followed up at 6 weeks, 12 weeks, and 24 weeks post completion of radiotherapy. The treatment planning process involved the following steps.

### A) The patient education and training regarding the procedure

The recruited patients were called for training 1 week prior to their CT simulation date. They were advised to practice breath hold for a minimum of 25 s. The importance of depth of respiration/chest expansion and the futility of abdominal respiration were mooted. The patient was asked to lie down in supine position in bed with upper torso at an elevated angle using pillow at home and to try breath hold for 5 s initially and then gradually increase the duration to 10, 15, 20, and 25 s.

### B) Patient treatment position, immobilization, and planning imaging

**Fig. 1:**
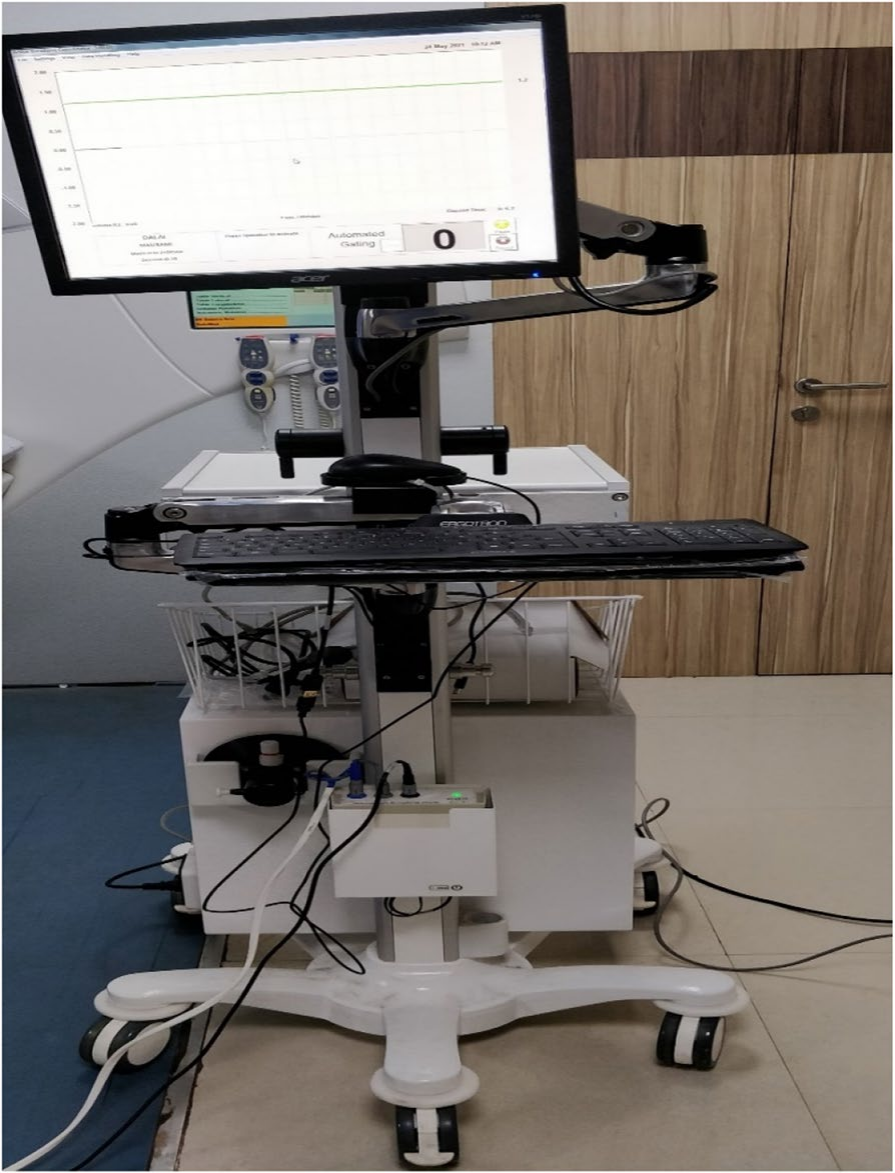
ABC system with the digital spirometer

**Fig. 2:**
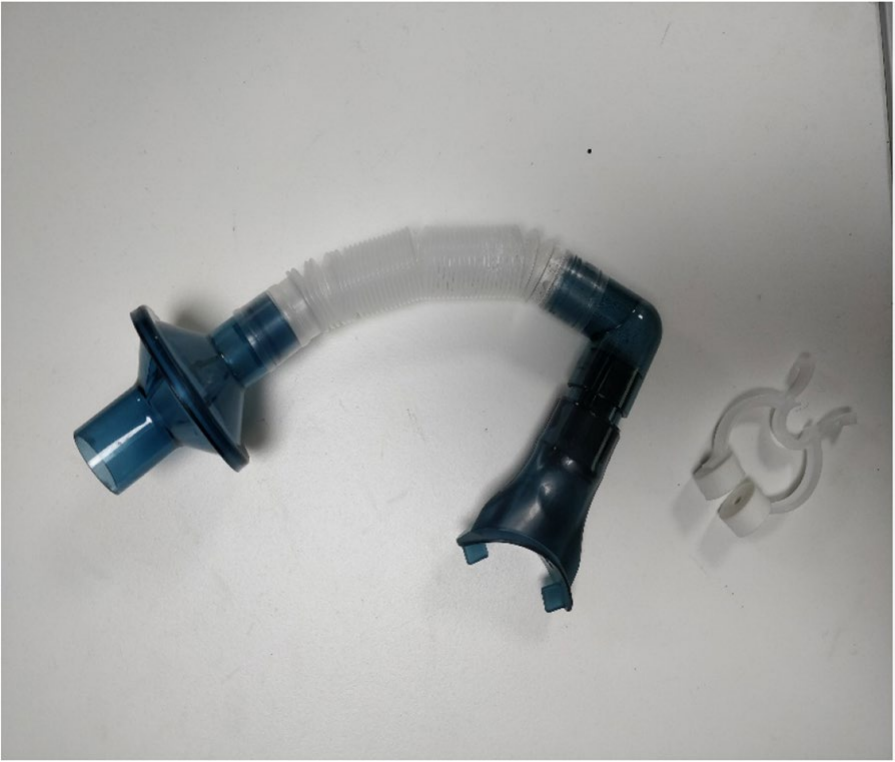
Mouthpiece and nose clip

**Fig. 3:**
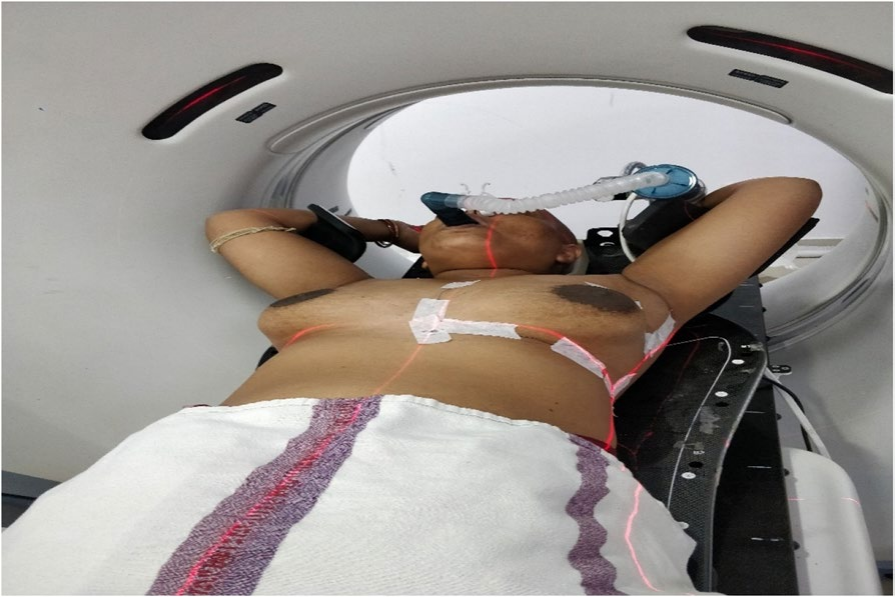
CT simulation using active breathing coordinator

Patient was positioned in proposed supine position on a breast board with 12.5-degree tilt. The arms were abducted, and the head was titled to the opposite side or kept straight. Nose clip was used to block the nares. Mouthpiece was kept inside the mouth which was connected to the spirometer (Figs. 1 and 2). Radiopaque markers were placed delineating mid-axillary line, midline of body and lower border of opposite breast, and mark repositioning lines on patient and immobilization devices. The volume of breath hold and breath hold duration are set in the system that is exclusively for ABC. The threshold volume is set as the 75% of the maximum inspiratory volume of the patient. The threshold duration was decided based on the patient’s preprocedure capacity to hold the breath. Three radio-opaque fiducials were kept one in midline and two on lateral surface for scan isocentre localization after the patient holds the breath to the predefined volume (Fig. 3).

The patient alignment was checked by taking topograms. Two volumetric non-contrast CT scans of the patient in treatment position were taken — one in moderate breath hold and another in free breathing. The extend of the scan was from lower body of mandible to L3 vertebrae. The slice thickness was 2.5 mm. The images were transferred to Monaco version 5.51.10 Elekta, Inc. three-dimensional treatment planning system (3DTPS).

### C) Target volume, organs at risk delineation, and treatment planning

RTOG contouring guidelines were followed to create the target volumes. Organs at risk (OAR) contouring — the OARs included for the purpose of study included the heart, the left anterior descending coronary artery, ipsilateral and contralateral lung, right breast, and spinal cord. To avoid inter-observer bias, contouring was done by the same physician in both the CT scans. Treatment planning was done by 3DCRT in both the CT scans.

### D) Dose prescription

Prescription dose for planning target volume (PTV) was 50 Gy in 25 fractions, 5 fractions per week over 5 weeks. High-Energy Linear Accelerator, Elekta Versa HD was used for treatment. The energy used was 6-MV photons for tangential fields and 6 MV/10 MV photons for supraclavicular fields by 3DCRT technique. Bolus was used for only tumours with skin involvement. Alternate-day treatment with bolus over chest wall was used wherever indicated. The dose–volume constraints for organs at risk were as follows: The treatment plan was to be accepted if 95% of dose covered > = 95% of PTV, if not achieved, at least 90% of dose covered > = 95% of PTV. The volume of ipsilateral lung receiving 20 Gy was ≤ 35% (i.e. V_20_ ipsilateral lung ≤ 35%) [7]. The volume of the heart receiving 25 Gy was tried to be kept ≤ 10% (i.e. V_25_ heart ≤ 10%) and spinal cord D_max_ (maximum point dose) < 45 Gy.

### E) Plan verification and execution

**Fig. 4:**
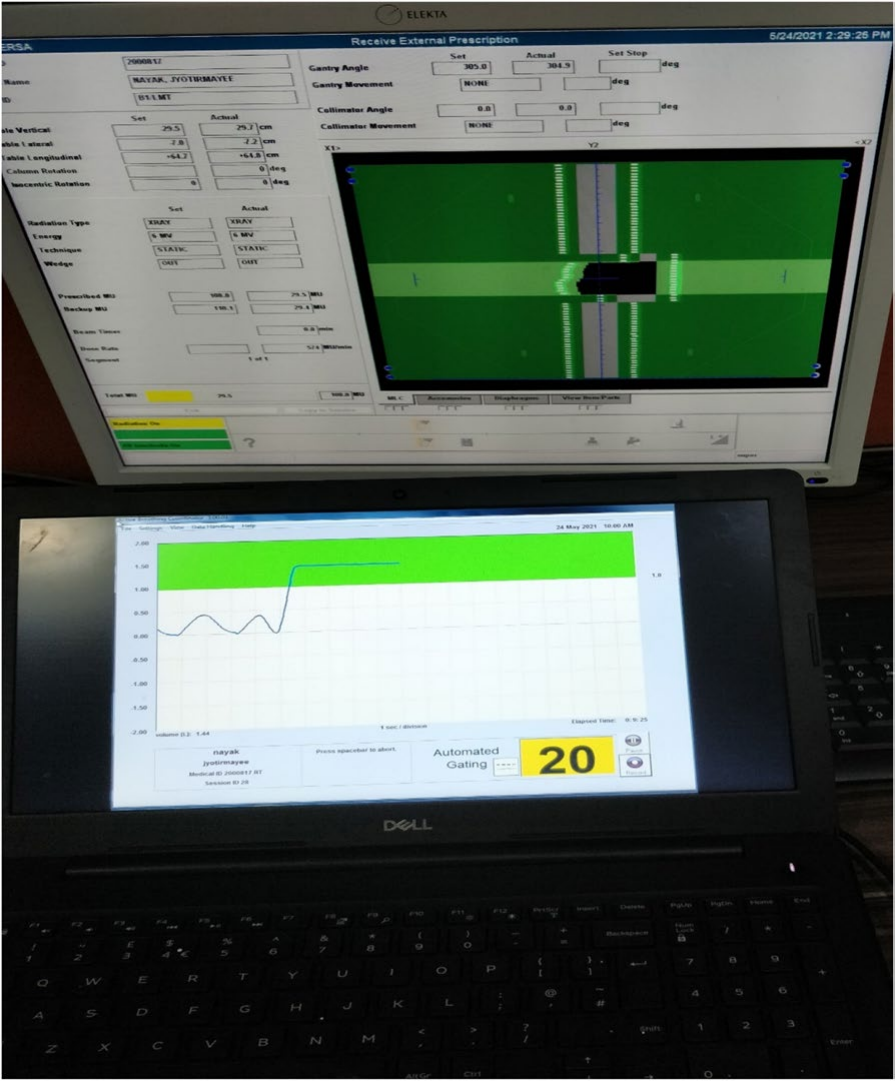
Treatment delivery using active breathing coordinator

Before execution of treatment plan, we have to setup the patient position with the help of external laser system provided in the machine room. The verification of the patient position is done by electronic portal imaging device (EPID). The main tangential fields can also be visualized, and this will ensure that the whole breast/chest wall is inside the treatment fields. This is done in the first three fractions and then biweekly. After ensuring the correct position of patient, treatment was delivered in ABC (Fig. 4).

Statistical analysis was done on IBM SPSS (Statistical Package for Social Sciences) version 26. The data on parameters were expressed as mean with standard deviation. The dosimetric profiles were compared using the paired *t*-test. All statistical analysis was carried out at 5% level of significance, and *p*-value < 0.05 was considered significant.

The study was passed by the institute ethics committee bearing number IEC/AIIMS BBSR/PG Thesis/2020-21/70.

## Results

A total of 32 patients were selected to be included in the study. The mean breath-hold time was 24 s. The mean breath-hold volume was 1.2 l. The mean time duration for CT simulation as well as for treatment for ABC was 20 min, while that for in FB was 10 min. The mean conformity indices of ABC and FB plans were 0.94.

The mean age of patients was 46.8 years, and median age was 46 years. A total of 62.50% of the patient were perimenopausal, while 18.75% were pre- and postmenopausal females. The patients with comorbidities constituted 34% of study cohort. Six patients had hypertension, three patients had diabetes mellitus, and seven had hypothyroidism. Four patients had more than one comorbidity (Table 1).

**Table 1:** Demographic characteristics

S. no	**Variable (*N* = 32)**	**No. of patients (percentage)**
1.	**Age (in years)**	46 years (median)
		22–64 years (range)
	< = 40 years	6 (18.75%)
	> 40 years to < = 55 years	20 (62.50%)
	> 55 years	6 (18.75%)
2.	**Comorbidities**	**No. of patients (percentage)**
	None	11 (34.37%)
	Hypertension	6 (18.75%)
	Diabetes mellitus	3 (9.37%)
	Hypothyroidism	7 (21.87%)

The median tumour size was 3.4 cm. The most common T stage was T2 (53.12%) followed by T4b (21.80%). The most common nodal stage was N0 and N1 (43.75% each). The most common stage encountered was stage IIA (37.50%) followed by IIIB (21.80%). Sixteen patients were positive for oestrogen receptor, while 16 were negative. Fourteen patients were positive for progesterone receptor, while 18 were negative. All hormone receptor-positive patients received hormone therapy (tamoxifen or letrozole) after completion of radiotherapy. Ten patients were positive for Her2/neu and received anti-HER2/neu therapy in the form of trastuzumab as neoadjuvant/adjuvant therapy (Table 2).

**Table 2:** Tumour characteristics

S. no	Variable (*N* = 32)	
1.	**Size (cm)**	**No. of patients (percentage)**
	Range: 1.4–7.0 cm	
	≤ 2	05 (15.62%)
	> 2 ≤ 5	20 (62.50%)
	> 5	07 (21.80%)
2.	**T stage (according to AJCC 8th edition)**	**No. of patients (percentage)**
	T1	4 (12.50%)
	T2	17 (53.12%)
	T3	3 (9.30 %)
	T4a	0 (0%)
	T4b	7 (21.80%)
	T4c	0 (0%)
	T4d	1 (3.12%)
3.	**N stage (according to AJCC 8th edition)**	**No. of patients (percentage)**
	N0	14 (43.75%)
	N1	14 (43.75%)
	N2a	2 (6.25%)
	N2b	1 (3.12%)
	N3a	0 (0%)
	N3b	1 (3.12%)
	N3c	0 (0%)
4	**Stage-wise distribution**	**No. of patients (percentage)**
	IA	3 (9.30%)
	IB	0 (0%)
	IIA	12 (37.50%)
	IIB	5 (15.62%)
	IIIA	4 (12.50%)
	IIIB	7 (21.80%)
	IIIC	1 (3.12%)
	IV	0 (0%)
5	**Molecular characteristics**	
i	**Oestrogen receptor**	**No. of patients (percentage)**
	Positive	16 (50%)
	Negative	16 (50%)
ii	**Progesterone receptor**	**No. of patients (percentage)**
	Positive	14 (43.75%)
	Negative	18 (56.25%)
iii	**HER2/neu receptor**	**No. of patients (percentage)**
	Positive	10 (31.25%)
	Negative	22 (68.75%)

*AJCC*, American Joint Committee on Cancer

Thirteen (40.62%) patients received neoadjuvant therapy. Three patients received three-weekly docetaxel of which one patient received trastuzumab too and eight patients received four cycles of three-weekly regimen (Adriamycin + cyclophosphamide) of which one patient received paclitaxel and one patient received paclitaxel and trastuzumab additionally. Two patients received weekly paclitaxel of which one patient received trastuzumab too. A total of 59.38% underwent upfront surgery. Sixteen patients each underwent modified radical mastectomy and breast-conserving surgery. Twenty-nine (90.62%) patients received adjuvant chemotherapy. Thirteen patients received Adriamycin and cyclophosphamide in adjuvant setting, while 12 patients got paclitaxel and 6 patients received docetaxel. Ten patients (31.25%) who were HER2/neu positive received anti-her2neu therapy trastuzumab in adjuvant setting (Table 3). Axillary and supraclavicular lymph nodal irradiation along with chest wall/whole breast was done in 21 patients, while 11 patients received whole breast irradiation only.

**Table 3:** Neoadjuvant/adjuvant treatment characteristics

S. no.	Variable (*N* = 33)	**Number (percentage)**
1.	**Neoadjuvant therapy**	
	Received neoadjuvant therapy	13 (40.62%)
	Did not receive neoadjuvant therapy	19 (59.37%)
2.	**Response to neoadjuvant chemotherapy**	**No. of patients (percentage)**
a	Clinical partial response	12 (92.30%)
b	Pathological complete response	0 (0%)
c	Disease progression	1 (7.6%)
i	Tumour progression	1 (7.6%)
ii	Lymph nodal progression	0 (0%)
2.	**Surgery**	**No. of patients (percentage)**
	Modified radical mastectomy	16 (50%)
	Breast conservation surgery	16 (50%)
3.	**Adjuvant chemotherapy**	**No. of patients (percentage)**
	Received	29 (90.62%)
	None	3 (9.30%)
4	**Her2 positive received anti-her2/neu therapy**	10 (31.25%)

**Fig. 5:**
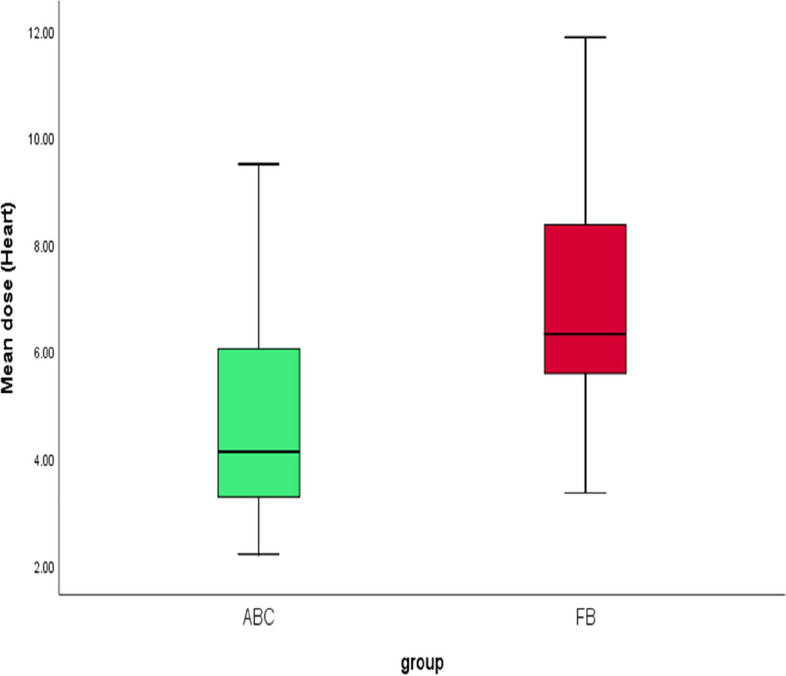
Box-Whisker plot showing the mean dose received by heart. ABC, active breathing coordinator. FB, free breathing

On comparison of dosimetric parameters of the heart between ABC and FB, all were found to be significantly lower in the ABC plans. There was a mean difference of 2.01 Gy between the ABC and FB mean heart doses corresponding to a 29% reduction in mean dose to the heart. V30 of the heart was 8.39% in FB, while it was 4.55% in ABC (Table 4) (Fig. 5).

**Table 4:** Dosimetric parameters of organs at risk

**Parameter**	**ABC**		**FB**		***p*-value**
	**Mean**	**SD**^**a**^	**Mean**	**SD**^**a**^	
**Heart**					
Dmean (Gy)	4.73	2.02	6.74	2.15	0.001
V5 (%)	14.54	6.79	20.41	6.30	0.001
V10 (%)	9.87	6.06	14.97	6.03	0.001
V30 (%)	4.55	3.87	8.39	4.50	0.001
**LADA**					
Dmean (Gy)	32.99	23.17	46.65	19.31	0.002
V30 (%)	19.32	8.47	24.87	8.31	0.001
**Left lung**					
Dmean (Gy)	13.20	3.03	14.80	3.30	0.001
V5 (%)	44.82	9.80	45.52	12.77	0.025
V10 (%)	34.56	8.67	37.53	8.92	0.001
V20 (%)	26.88	7.39	30.31	8.03	0.001
**Right lung**					
Dmean (Gy)	0.74	0.14	0.81	0.16	0.004
V5 (%)	0.08	0.12	0.14	0.21	0.086
**Right breast**					
Dmean (Gy)	1.07	0.38	1.17	0.26	0.025
V5 (%)	0.91	1.63	1.16	1.2	0.102
Dmax (Gy)	17.85	13.34	22.96	13.52	0.084

^a^Standard deviation

**Fig. 6:**
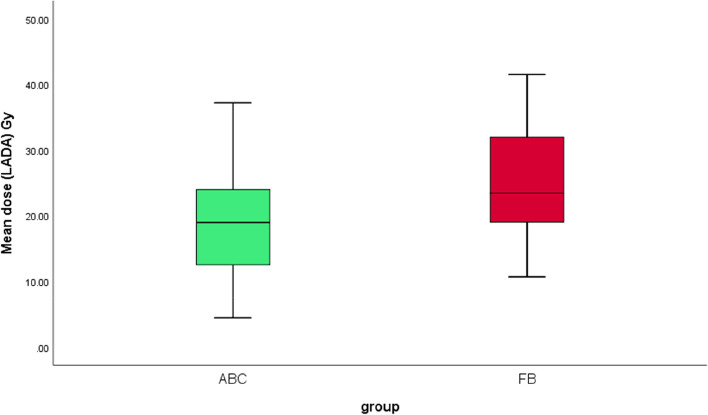
Box-Whisker plot showing the mean dose received by left anterior descending coronary artery. LADA, left anterior descending coronary artery. ABC, active breathing coordinator. FB, free breathing

The mean dose and V30 of LADA were 32.99 Gy and 19.32, respectively, in ABC plans. In FB plans, mean dose and V30 were 46.65 Gy and 24.87%, respectively. The difference in mean dose was 13.66 Gy which corresponds to a 29.28% reduction in dose to LADA (Table 4) (Fig. 6).

The mean dose to the left lung was 13.2 Gy in ABC plan, while it was 14.80 with FB plan. The V5, V10, and V20 of the left lung were also lower in ABC plan (44.82% vs 45.52%, 34.56% vs 37.53%, 26.88% vs 30.31%, ABC vs FB, respectively) (Table 4).

The mean dose to the right lung was also lower in ABC plans as compared to FB plans and was statistically significant too (0.74 Gy vs 0.81 Gy, respectively) with a *p*-value of 0.004. But the V5 was comparable between the two groups with numerically lower value in the ABC group (0.08% vs 0.14%, ABC vs FB, *p*-value = 0.086) (Table 4).

All the parameters of the left lung (Dmean, V5, V10, V20) were significantly higher in the 21 patients who received axillary and supraclavicular irradiation (in ABC and free-breathing plans). The rest of the parameters of the heart, LADA, right lung, and right breast was not significantly different between the groups (Table 5). Similarly, patients receiving radiotherapy along with a boost dose tumour bed did not have any dosimetry difference between free breathing and ABC with respect to heart and lung doses (Table 6).

**Table 5:** Dosimetric parameters of the lung and heart with regional nodal irradiation (RNI) between ABC and free breathing

Left lung	RNI (*n* = 21) FB	No RNI (*n* = 11) FB	*p*-value	RNI (*n* = 21) ABC	No RNI (*n* = 11) ABC	*p*-value
Dmean (Gy)	16.61 ± 2.26	11.65 ± 2.41	.001	14.47 ± 2.37	10.82 ± 2.61	.001
V5 (%)	52.49 ± 6.31	33.43 ± 12.83	.001	49.50 ± 7.01	36.88 ± 9.42	.001
V10 (%)	42.40 ± 6.11	29.17 ± 6.95	.001	38.68 ± 0.662	27.47 ± 7.35	.001
V20(%)	34.60 ± 5.94	22.94 ± 5.70	.001	30.15 ± 5.99	21.05 ± 5.85	.001
Heart						
Dmean (Gy)	7.12 ± 2.02	6.40 ± 2.65	0.44	4.66 ± 1.98	4.76 ± 2.10	0.89
V5 (%)	21.61 ± 6.15	19.29 ± 7.49	0.39	14.35 ± 6.64	14.52 ± 7.20	0.94
V10 (%)	16.32 ± 5.72	13.48 ± 7.20	0.37	10.07 ± 5.77	9.23 ± 6.63	0.72
V30 (%)	9.20 ± 4.10	7.58 ± 5.60	0.40	4.63 ± 3.79	4.19 ± 4.06	0.76
LADA						
Dmean (Gy)	25.20 ± 7.76	24.97 ± 9.64	0.94	20.69 ± 8.73	16.65 ± 7.12	0.17
V30 (%)	46.91 ± 17.36	47.74 ± 23.34	0.91	36.30 ± 24.26	25.71 ± 19.06	0.18

**Table 6:** Dosimetric parameters of the lung and heart with or without additional boost doses between ABC and free breathing

Left lung	Boost (*n* = 16) ABC	No boost (*n* = 16) ABC	*p*-value	Boost (*n* = 16) FB	No boost (*n* = 16) FB	*p*-value
Dmean (Gy)	11.75 ± 2.78	14.68 ± 2.47	.004	12.98 ± 3.10	16.83 ± 2.22	.001
V5 (%)	41.08 ± 10.38	49.30 ± 7.63	.016	42.41 ± 9.95	49.47 ± 14.55	0.129
V10 (%)	31.02 ± 8.62	38.63 ± 7.04	.010	33.20 ± 9.07	42.50 ± 6.13	.002
V20(%)	23.73 ± 6.92	30.32 ± 6.29	.008	26.51 ± 7.93	34.68 ± 5.97	.003
Heart						
Dmean (Gy)	4.40 ± 1.93	5.00 ± 2.07	0.40	6.35 ± 2.25	7.39 ± 2.18	0.19
V5 (%)	13.60 ± 6.79	15.21 ± 6.78	0.50	19.58 ± 6.85	22.05 ± 6.35	0.30
V10 (%)	8.64 ± 6.02	10.92 ± 5.92	0.28	13.92 ± 6.46	16.77 ± 6.01	0.20
V30 (%)	3.59 ± 3.56	5.37 ± 3.98	0.19	7.53 ± 4.62	9.97 ± 4.54	0.18
LADA						
Dmean (Gy)	17.32 ± 8.10	21.28 ± 8.33	0.19	23.54 ± 8.02	26.71 ± 8.52	0.41
V30 (%)	27.34 ± 22.23	37.98 ± 22.95	0.18	44.39 ± 20.68	50 ± 17.90	0.28

The dosimetry of right breast was comparable between the two groups with respect to V5 and maximum dose (0.91% vs 1.16%, 17.85 Gy vs 22.96 Gy, ABC vs FB). But the mean dose received by right breast was significantly lower in the ABC group (1.07 Gy vs 1.17 Gy, *p*-value = 0.025) (Table 4).

## Discussion

Breast cancer is the most common cancer diagnosed worldwide in both sexes, combined and women [1].

Long-term toxicity of breast cancer radiotherapy includes cardiac and lung side effects. Breast oedema, radiation fibrosis, and brachial plexopathy can also be present, but longevity is affected little. Cardiac side effects such as myocardial infarction, ischaemic heart disease, and radiation-induced heart disease can impact the patient’s overall survival. Ironically, cancer treatment rather than cancer determines the patient’s survival in this scenario. So, attempts at reducing the dose received by the heart have been the point of interest of researchers in the past two decades. Various modalities were tried, and ABC is one of the promising tools in place.

### Patient selection

Various studies have attempted optimal patient selection for ABC. Ryohei Yamauchi et al. [8] reported that a relative reduction of the mean heart dose correlated with the patient’s body mass index (BMI) DIBH was found to be more beneficial in the patients with low BMI. Soujanya Ferdinand et al. [9] correlated heart volume in field and maximum heart depth with decreased heart dose using ABC. The maximum heart depth was defined as the distance from the heart border to the edge of the field, while the heart volume in field was the volume of the heart within the 50% isodose line. In this study, a single parameter that predicted the smooth running of this programme is the ability of the patient to hold their breath. This depends on the ability of the patient to comprehend the instructions and, in turn, the practice. This was an observation echoed in the study by Eldredge Hindy et al. [10] Gabrielle Peters et al. [11] tried ABC for right breast cancer with regional lymph nodal irradiation. The lung (mean lung dose, V20, V5) and liver (Dmax, V20, V30) parameters decreased significantly with the use of ABC. But heart parameters did not improve significantly.

### Dosimetric parameters of the heart

In this study, the dosimetric parameters of the heart, such as the mean dose, V5, V10, and V30, were compared between the ABC and FB plans. All the parameters were significantly lower in the ABC set of plans. Similar results were seen in the study by Beena Kunheri et al. [12], where parameters such as V5, V10, V15, V20, V25, V30, V35, V40, Dmean, and Dmax were compared. All the values were lower in the ABC group with statistical significance. In a 6-year data by Swanson et al. [13], there was a significant improvement in heart dose parameters with ABC treatment (V5, V10, V15, V20, V25, V30, MHD). A percentage reduction of the dose by 40% was observed. A systematic review by Lloyd M. Smith et al. [14] of the literature based on DIBH versus FB dosimetric comparison showed a percentage reduction in mean heart dose to the range of 38 to 67%. In this study, the reduction was to the tune of 29.8%.

### Dosimetric parameters of lung

The lung dose received by the patients treated with ABC was also significantly lower with respect to Dmean, V5, V10, and V20. These results concurred with the findings by Eldredge Hindy et al. [10]. Except for the maximum dose, other parameters of the left lung, such as mean dose, V5, V10, and V20, were found to be lower with ABC. The total lung parameters were also significant except for V5. In this study, V5 of the right lung was lower but not statistically significant even though the mean doses were lower and significant in the ABC group. The study by Ferrat Dincoglan et al. [15] showed significantly lower values for ipsilateral and lung parameters, such as V20 and Dmean. V5 was not reported here. On the contrary, V5, V10, V20, and mean doses of left lung and whole lungs were significantly lower in the study by Lin et al. [16].

### Dosimetric parameters of the right breast

In this study, the mean dose to the right breast was found to be significantly lower in the ABC group. But V5 and Dmax were comparable. In fact, very few studies have attempted to study the right breast dosimetry. Ferrat Dincoglan et al. [15] reported a reduction in both the maximum and the mean right breast doses.

### Dosimetric parameters of LADA

There was a significant reduction in the mean dose as well as V30 of LADA. There was an absolute difference of 13.66 Gy in the mean dose received by LADA with ABC. As the tangential fields tend to include the left ventricle, LADA invariably receives the major brunt. Dosimetry of LADA was not routinely considered in earlier studies. The study by Beena Kunheri et al. [12] showed a 53% reduction of mean dose to LADA using ABC. Regardless of the kind of DIBH used, a systematic review by Smyth et al. [14] had shown a consensus of reduction in mean LADA dose to a range of 31 to 71%. In this study, a reduction of 29.28% was achieved.

The variation in results and findings of DIBH plans versus free-breathing plans may be attributed to the wide spectrum of eligibility criteria employed in various studies, the technique of radiotherapy attempted (IMRT vs 3DCRT), the technique of DIBH (RPM vs ABC), and the difference in breast volumes between races. But in most of the cases, it was evident that there is a clear benefit of using ABC-mDIBH in breast cancer, especially left sided for cardiac and ipsilateral lung sparing.

### Drawbacks of the study

Clinical implications of the improvements in dosimetry regarding the cardiac and respiratory were not assessed as a part of the study as long-term follow-up is required. All subsets of left breast cancer patients were included in the study. These subsets include the patients who underwent BCS and MRM and early and locally advanced cases. It is challenging to know which subset of these patients received the maximum benefit from using ABC. The numerical advantage of dosimetry was seen more in MRM patients than in BCS. The study also did not explore the possibility of finding other factors that may predict better dosimetric outcomes, such as the mean heart distance, lung distance, and mean heart volume. 3DCRT was the technique that was used for all the plans. The impact of using IMRT, VMAT, or hybrid IMRT is areas that need further research.

## Conclusion

This study compared the dosimetry of ABC-mDIBH to that of free breathing concerning the heart, LADA, right lung, left lung, and right breast. All the dosimetric parameters of the heart, such as the mean dose, V5, V10, and V30, improved with plans under ABC. The parameters of LADA followed suit. The mean dose and V30 were significantly higher with free breathing. Concerning the left lung, mean dose, V5, V10, and V20 were significantly lower when treated under ABC. Even though numerically lower, the V5 of the right lung, V5, and maximum dose of the right breast were comparable between the ABC and FB groups. At the same time, mean doses received by the right breast and right lung were significantly lower with ABC.

If there are no respiratory disorders and if the patient can comprehend the instructions, ABC-mDIBH should be considered for all left breast cancer cases. With proper training, better inspiratory volumes and the resultant improvement in the quality of plans can be achieved. Thus, ABC should be the routine method of adjuvant radiation in left breast cancer cases.

## Data Availability

The data sets used/analysed during the current study are available on Figshare: https://doi.org/10.6084/m9.figshare.24899826.v1

## Abbreviations

mDIBH: Moderate deep inspiratory breath hold
FB: Free breathing
ABC: Active breathing coordinator
Vx: Volume of an organ receiving a dose of ‘x’ Gy
LADA: Left anterior descending coronary artery
3DCRT: Three-dimensional conformal radiotherapy
CT: Computed tomographic scan
PTV: Planning target volume
